# GSK‐3β inhibition protects the rat heart from the lipopolysaccharide‐induced inflammation injury via suppressing FOXO3A activity

**DOI:** 10.1111/jcmm.14656

**Published:** 2019-09-10

**Authors:** Zhigang Li, Huifang Zhu, Chang Liu, Yumei Wang, Duo Wang, Huan Liu, Wenze Cao, Yi Hu, Qin Lin, Chang Tong, Min Lu, Agapios Sachinidis, Li Li, Luying Peng

**Affiliations:** ^1^ Key Laboratory of Arrhythmias Ministry of Education Shanghai East Hospital Tongji University School of Medicine Shanghai China; ^2^ Research Center for Translational Medicine Shanghai East Hospital Tongji University School of Medicine Shanghai China; ^3^ Institute of Neurophysiology and Center for Molecular Medicine, Cologne (CMMC) University of Cologne Cologne Germany

**Keywords:** cardiac dysfunction, FOXO3A, GSK‐3β, inflammation injury, NF‐κB, sepsis

## Abstract

Sepsis‐induced cardiac dysfunction represents a main cause of death in intensive care units. Previous studies have indicated that GSK‐3β is involved in the modulation of sepsis. However, the signalling details of GSK‐3β regulation in endotoxin lipopolysaccharide (LPS)‐induced septic myocardial dysfunction are still unclear. Here, based on the rat septic myocardial injury model, we found that LPS could induce GSK‐3β phosphorylation at its active site (Y216) and up‐regulate FOXO3A level in primary cardiomyocytes. The FOXO3A expression was significantly reduced by GSK‐3β inhibitors and further reversed through β‐catenin knock‐down. This pharmacological inhibition of GSK‐3β attenuated the LPS‐induced cell injury via mediating β‐catenin signalling, which could be abolished by FOXO3A activation. In vivo, GSK‐3β suppression consistently improved cardiac function and relieved heart injury induced by LPS. In addition, the increase in inflammatory cytokines in LPS‐induced model was also blocked by inhibition of GSK‐3β, which curbed both ERK and NF‐κB pathways, and suppressed cardiomyocyte apoptosis via activating the AMP‐activated protein kinase (AMPK). Our results demonstrate that GSK‐3β inhibition attenuates myocardial injury induced by endotoxin that mediates the activation of FOXO3A, which suggests a potential target for the therapy of septic cardiac dysfunction.

## 1 INTRODUCTION

Sepsis, a complex syndrome with an enormous societal burden in terms of cost, morbidity and mortality, is a severe infection caused by Gram‐negative bacteria, which could affect virtually every organ including heart, kidney, liver and lung.^1^, ^2^, ^3^ Among all of the sepsis‐induced injuries, cardiac dysfunction is one of the most severe manifestation. Growing evidence suggests that cardiac dysfunction by sepsis increases the risk of mortality rates up to 70%‐90%, while patients without cardiac dysfunction experience less than 20% mortality rate.^4^ Some findings propose possible causes of septic‐induced cardiomyopathy, including uncontrolled inflammatory responses, myocardial and mitochondrial energy metabolism disorders and apoptosis.^5^, ^6^

GSK‐3β is a multifunctional serine/threonine kinase involved in regulating cell fate and differentiation processes in a variety of organisms^7^ and can exert a central role in diverse signalling pathways such as Wnt, Notch, Hedgehog, NF‐κB and AMPK.^8^, ^9^ Recently, GSK‐3β has been identified as a crucial regulator in modulation of inflammation,^10^, ^11^ in which the knock‐down of GSK‐3β diminishes LPS‐associated pro‐inflammatory cytokines and significantly reduces the mortality of human monocytes.^10^ The best characterized upstream kinase that inactivates GSK‐3β through phosphorylation at ser‐9 is Akt, which is well established as a protective effector in sepsis.^12^ Similar to GSK‐3β, Akt also directly attenuates the functions of FOXO3A through phosphorylation modification, and its inhibition causes FOXO3A activation.^13^

FOXO3A is a member of FOXO transcription factor family consisting of FOXO1, FOXO3, FOXO4 and FOXO6, and mainly participates in the transcriptional regulation of autophagy, inflammation, apoptosis and cell cycle arrest.^14^, ^15^, ^16^ Increasing evidence indicates that FOXO3A modulates inflammation through regulating NF‐κB,^17^ but its role in endotoxin‐induced myocardial injury still remains unclear. It has been showed that GSK‐3β positively regulates hepatoma cell proliferation through the transactivation activity of FOXO3A,^18^ how about the cross‐talking between FOXO3A and GSK‐3β in endotoxin‐induced heart injury has not been explored yet. FOXO3A could trigger cell apoptosis through promoting the expression of pro‐apoptotic transcription factors such as Bim, PUMA,^19^ Tradd^20^ and Mxi1‐0.^21^ Hereby, we hypothesize that GSK‐3β inhibition may protect against endotoxin‐induced myocardial injury via FOXO3A‐dependent mechanisms.

In the present work, we found that pharmacological inhibition of GSK‐3β preserved cardiac ejection fraction (EF) and fractional shortening (FS) in LPS‐treated rats. The inhibition of GSK‐3β reduced LPS‐induced apoptosis in cardiomyocytes. FOXO3A was identified to be positively regulated by GSK‐3β through degrading β‐catenin, and the knock‐down of FOXO3A in cardiomyocytes with siRNAs further confirmed its positive role on sepsis‐induced cardiomyocytes dysfunction. These data indicate that both GSK‐3β and β‐catenin participate in the pathogenesis of endotoxin‐induced cardiac dysfunction via activation of FOXO3A.

## 2 MATERIALS AND METHODS

### 2.1 Animals

All the rats were purchased from Slaccas Company (Shanghai, China) and kept in the animal facility at Tongji University. All of the procedures were approved by Institutional Animal Care and Use Committee at Tongji University (Approval No: TJLAC‐016‐022). All of animal experiments were performed in accordance with the National Institutes of Health guide for the care and use of Laboratory animals.

### 2.2 Endotoxemia model

All experiments were operated on male Sprague Dawley rats that weighed between 220 g and 250 g. Rats were randomly divided into four groups: sham group, endotoxin group, negative group and positive group. Endotoxemia was induced by intraperitoneal (ip) injection with 4 mg/kg LPS (Sigma). Negative controls or positive groups were pre‐treated with 100 mg/kg NaCl (Sigma) or with 100 mg/kg LiCl (Sigma), respectively, 3 days before or after LPS injection. 6 hours after stimulation with LPS, cardiac functions were evaluated and samples from each group were collected. All rats were killed under deep anaesthesia with isoflurane (Gene&I).

### 2.3 Cell culture and treatment

Neonatal Sprague Dawley rats were purchased from Slaccas Company (Shanghai, China). Primary newborn rat's cardiac myocytes (CMs) were isolated and cultured as previously described.^22^ Briefly, cells were digested with 0.1% type II collagenase (Invitrogen) and 0.1% trypsin (Gibco) mixture with gently shaking. Cells were resuspended with DMEM including 10% FBS (Gibco) and cultured at 37°C with 5% CO_2_ for 1 hour. Non‐adherent cells (myocytes) were collected and cultured in fresh medium (Gibco) harbouring 0.1 mM BrdU (sigma) for 48 hours. To induce sepsis, CMs were treated with LPS at different concentrations (0.1, 0.2, 0.5, 1.0 and 2.0 μg/mL) and time‐points (3, 6, 12, 24 and 48 hours). The GSK‐3β inhibitors LiCl (sigma) and CHIR‐99021 (Selleck) were added into CMs during culture for stimulation.

### 2.4 Western blot

CMs were lysed directly in the culture plate on ice with RIPA lysis buffer containing protease inhibitor cocktail (Roche), and tissues were also grinded with the same condition. Then, the lysates were centrifuged to remove any debris, and protein concentrations were measured using the BCA protein assay kit (TaKaRa). Protein samples were resolved using SDS‐PAGE gel and then transferred to 0.45 μm Immobilon‐PVDF membranes (Millipore). Primary antibodies used included cleaved‐caspase3 (Abcam, ab49822), NF‐κB (Abcam, ab16502), Bcl‐2 (Abcam, ab59348), GSK‐3β (Abcam, ab2602), p‐GSK‐3β (Abcam, ab75745), FOXO3A (Abcam, ab17026), β‐catenin (Abcam, ab32572), ERK (Cell Signaling Technology, CST4695), Bim (Cell Signaling Technology, CST2933T), p‐ERK (Cell Signaling Technology, CST4370), AMPK (Cell Signaling Technology, CST2532) and p‐AMPK (Cell Signaling Technology, CST2531). GAPDH (Proteintech, 60004‐1‐Ig) was applied as a loading control. The membranes were then incubated with secondary antibodies and imaged with ImageQuant LAS 4000 or Amersham Imager 600.

### 2.5 Quantitative real‐time PCR

The total RNA was isolated from tissues or cells with TRIzol reagent (Invitrogen), and cDNA was synthesized using PrimeScript™ RT reagent Kit with gDNA Eraser (TaKaRa) followed by a gene expression assay applying TB Green™ Premix Ex Taq™ (TaKaRa) on a Bio‐Rad CFX Connect™ real‐time system. The related primers were listed in Table S1.

### 2.6 Immunofluorescence staining

Cells were seeded on glass slides in 48‐well plate. After treatment, CMs were fixed with 4% paraformaldehyde in PBS for 15 minutes at room temperature (RT) and then permeabilized with 0.1% triton X‐100 before blocked with 10% goat serum for 1 hour. Primary antibodies (1:200) were incubated at 4°C overnight, following by fluorescent secondary antibodies (1:250) for 1 hour and Hoechst for 12 minutes at RT. Images were taken using Leica confocal microscope after blocking with aqueous mounting medium.

### 2.7 TUNEL assay

CMs apoptosis was detected by terminal deoxynucleotidyl transferase (TdT)‐mediated dUTP nick end labelling (TUNEL) assay. After fixed and permeated, each sample was treated with 80 μL of TUNEL reaction mixture for 60 minutes at 37°C in dark, and then, the samples were incubated with Hoechst for 12 minutes at RT, following by rinsing with PBS three times, and finally imaged using Leica confocal microscope.

### 2.8 siRNAs for β‐catenin and FOXO3A

Cells were seeded at 2 × 10^6^ in 6‐well plate or at 4 × 10^4^ in 24‐well plate. After growing for 48 hours, siRNA was transfected using Lipofectamine 2000 (Invitrogen) with a terminal concentration of 50 nM. 4‐6 hours later, cells were treated by changing fresh medium and cultured for another 24‐48 hours before conducting next experiments. The siRNA sequences were as following: si‐FOXO3A# CUCUAUAACGUAUGCAAAU, si‐β‐catenin#1 GCUGACCAAACUGCUAAAU and si‐β‐catenin#2 GCACCAUGCAGAAUACAAA. All of the siRNAs and control siRNA were synthesized by GenePharma as previously reported.^23^, ^24^

### 2.9 Separation of nuclear and cytoplasmic protein

Nuclear and cytoplasmic proteins were separated using NE‐PER™ Nuclear and Cytoplasmic Extraction Reagents kit (Thermo Scientific) according to the manufacturer's protocol. Proteinase inhibitors were added to each buffer immediately before use. Cells were harvested by scraping and rinsing with ice‐cold PBS, and then, the cells were lysed in ice‐cold CER I buffer for 15 minutes, following by adding ice‐cold CER II buffer and incubating on ice for 1 minute. Cell suspensions were centrifuged at 16 000 *g* for 5 minutes, and the supernatant containing the cytoplasmic proteins was collected for next experiments. The precipitation was resuspended with ice‐cold NER buffer and incubated on ice for 40 minutes. The samples were centrifuged at 16 000 *g* for 10 minutes, and the nuclear protein was collected and stored at −80°C for further use.

### 2.10 Echocardiography

After 6 hours of the treatment with LPS or saline through ip injection, rats were anaesthetized with isoflurane (3.0% induction in room air, followed with 0.5% maintenance in room air) and subjected to echocardiography using Vevo770 (Visual Sonics Inc) as previously described.^22^ The M‐mode images of left ventricular (LV) dimensions were obtained. The left ventricular EF (%) and FS (%) were measured, respectively. Echocardiography data were recorded and analysed individually.

### 2.11 Wheat germ agglutinin staining

Cardiomyocyte size was evaluated using wheat germ agglutinin staining. The rat heart was fixed in 4% paraformaldehyde, and then, the frozen tissues were sectioned into 20 μm slides, rinsed with PBS and stained for cardiomyocyte membrane with FITC‐conjugated wheat germ agglutinin (Sigma). Finally, the heart cross section was imaged with Leica confocal microscope.

### 2.12 Statistical analysis

ANOVA test was used to compare among three or more groups, followed by Bonferroni's post hoc test. Student's *t* test was applied to compare two groups, and the error bar represented the standard error of mean (SEM). A value of *P* < .05 was considered significant. All data were analysed using Prism 5.0 (GraphPad Software, Inc).

## 3 RESULTS

### 3.1 LPS induces activation of GSK‐3β in rat cardiomyocytes

**Figure 1:**
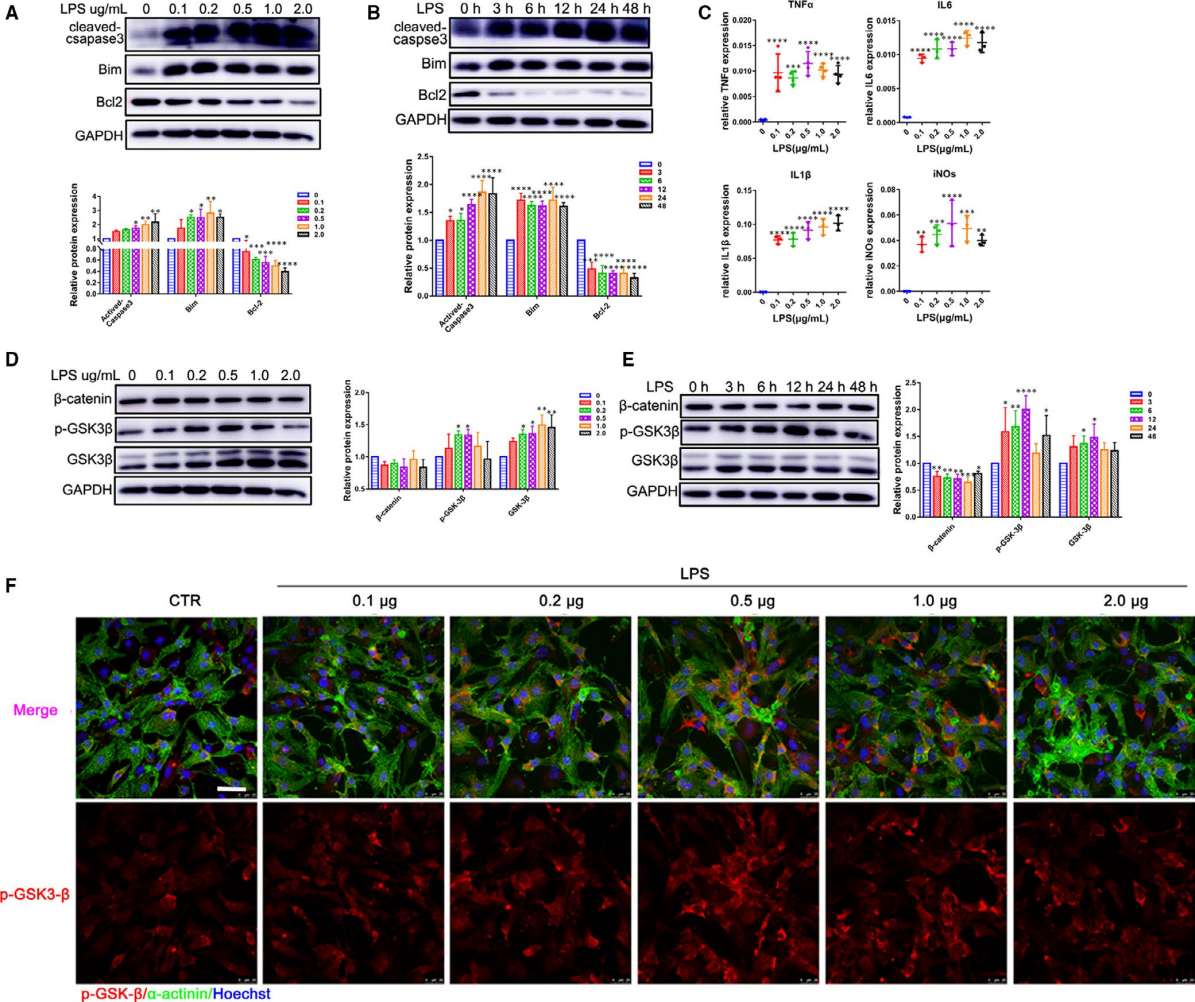
LPS induces inflammation injury and up‐regulates GSK‐3β in cardiomyocytes. A, B, CMs were treated with LPS (12 h) for different concentrations and stimulated with LPS (0.5 μg/mL) for different time. Western blot analysis for apoptosis‐related genes cleaved‐caspase3, Bim and Bcl‐2 expression (n = 3). C, qRT‐PCR analysis for the cytokines TNF‐α, IL‐1β, IL‐6 and iNOs (n = 3‐4). D, E, β‐catenin, GSK‐3β and p‐GSK‐3β (Y216) expression were measured by Western blot in CMs (n = 3). F, Immunofluorescence analysis for p‐GSK‐3β (Y216) and its location (n = 3). (Scale bar: 25 μm). *P < .05; **P < .01; or ***P < .001 and ****P < .0001 when compared with controls

Uncontrolled inflammation and apoptosis are two main features of endotoxin‐induced cardiac dysfunction.^10^, ^25^ Here, we examined the apoptosis rate of CMs exposed to LPS at different concentrations and incubation time. Our results showed a concentration‐dependent increase for the expression of the pro‐apoptosis proteins, cleaved‐caspase3 and Bim after treatment of the CMs with LPS for 24 hours. However, the expression of anti‐apoptosis gene Bcl‐2 was significantly decreased (Figure 1A). Furthermore, the expression of cleaved‐caspase3, Bim and Bcl‐2 protein was elevated in presence of LPS (Figure 1B). We then investigated the inflammatory response in CMs under different concentrations of LPS. The results displayed that LPS significantly increased the release of pro‐inflammatory cytokines IL‐6, IL‐1β and TNF‐α. Meanwhile, LPS treatment also promoted the mRNA expression of the chemotactic cytokine, iNOs (Figure 1C).

GSK‐3β can either positively or negatively affect a variety of transcription factors that are critical in regulating pro‐ and anti‐inflammatory cytokine production as well as cell survival.^26^ Therefore, we initially determined whether LPS could regulate the expression of GSK‐3β. To this end, CMs were treated for 12 hours by different concentrations of LPS (0.1, 0.2, 0.5, 1.0 and 2.0 μg/mL). Protein expression of GSK‐3β was up‐regulated as a concentration‐dependent manner rather than a stimulating‐time manner (Figure 1D,E). Interestingly, phosphorylation of GSK‐3β at Y216 showed a peak in the presence of 500 ng/mL LPS for 12 hours (Figure 1D‐F), which might be related to the potential role GSK‐3β at the early stage of inflammation injury.

### 3.2 GSK‐3β inhibition attenuates LPS‐induced cardiac inflammation injury

**Figure 2:**
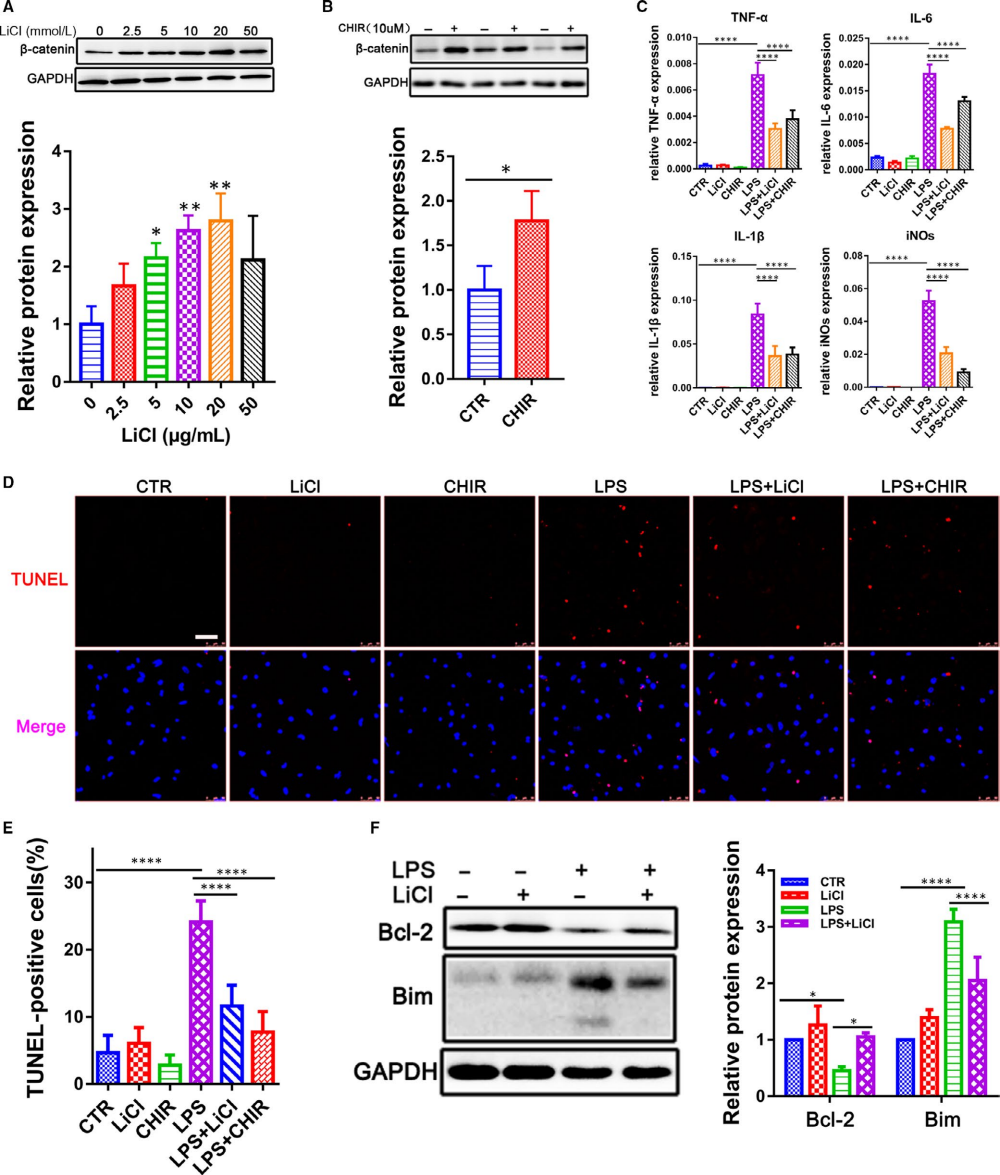
Down‐regulation of GSK‐3β attenuates myocardial inflammatory injury in cardiomyocytes after LPS challenge. A, B, Relative β‐catenin expression was measured by Western blot in the CMs treated with GSK‐3β inhibitors LiCl (0‐50 mM) and CHIR‐99021 (10 μM) for 24 h (n = 3). C, qRT‐PCR analysis for pro‐inflammatory cytokines TNF‐α, IL‐1β, IL‐6 and iNOs in CMs treated with LiCl (10 mM) and CHIR‐99021 (10 μM) in the presence of LPS (500 ng/mL) for 12 h (n = 3). D, E, TUNEL assay for apoptosis in CMs treated with LiCl (10 mM) and CHIR‐99021 (10 μM) in the presence of LPS (500 ng/mL) for 12 h. Nuclei were counterstained with the DNA‐intercalating dye Hoechst (blue). The lower panel was the percentage of TUNEL‐positive cells (n = 3). (Scale bar: 25 μm). F, Western blot for Bim and Bcl‐2 in CMs pre‐treated with LiCl (10 mM) for 12 h and followed by stimulation of LPS (500 ng/mL) for another 12 h. GAPDH was loaded as internal control (n = 3). *P < .05; **P < .01 and ****P < .0001 when compared with controls

CHIR‐99021 and LiCl were widely used as GSK‐3β inhibitors in a variety of cell types.^10^ We applied CHIR‐99021 and LiCl to CMs to assess their potential inhibition on GSK‐3β. β‐catenin was used as an indicator of GSK‐3β activity because β‐catenin could be phosphorylated and degraded by GSK‐3β. Our results showed that CHIR‐99021 and LiCl could significantly increase the β‐catenin level (Figure 2A,B), which demonstrated their ability to inhibit GSK‐3β activity in CMs.

To investigate whether GSK‐3β played a critical role in LPS‐induced CMs inflammation injury, we treated the CMs with LPS in the presence and absence of CHIR‐99021 and LiCl. We found that the GSK‐3β inhibition decreased the LPS‐induced activation of pro‐inflammatory cytokines IL‐6, IL‐1β, TNF‐α and iNOs (Figure 2C). Moreover, during the LPS‐induced inflammatory response, CHIR‐99021 and LiCl could significantly reduce the TUNEL‐positive cell numbers in CMs (Figure 2D,E). It has been showed that Bcl‐2, as an indicator of anti‐apoptosis, can suppress cell death through inhibiting Bim. As expected, the inhibition of GSK‐3β could increase the expression of Bcl‐2 but decrease the expression of Bim (Figure 2F), suggesting that GSK‐3β may aggravate cardiac dysfunction and promote apoptosis in LPS‐stimulated rats.

### 3.3 GSK‐3β inhibition improves cardiac function in vivo

**Figure 3:**
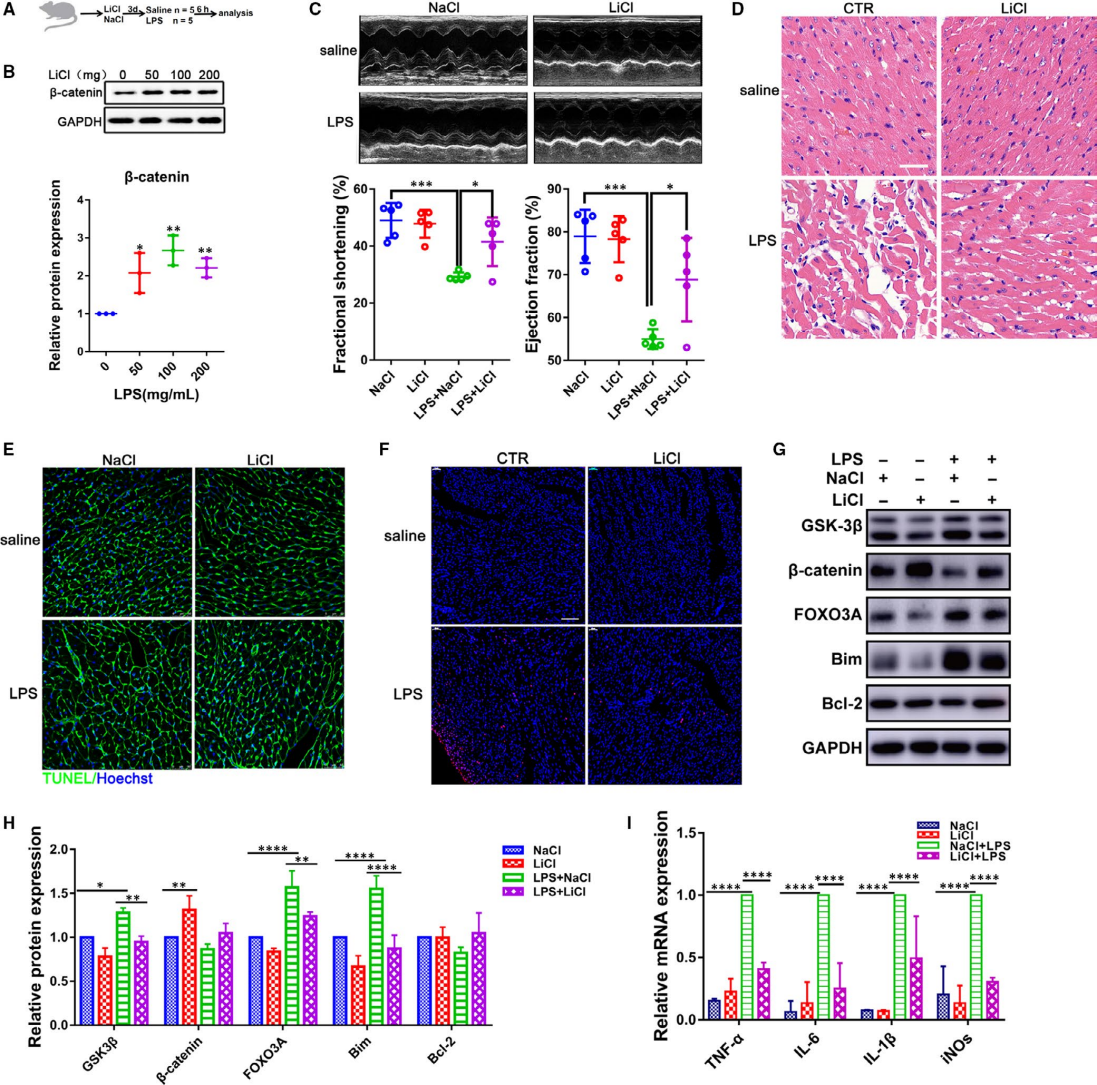
Down‐regulation of GSK‐3β improves cardiac function and abrogates apoptosis in LPS‐treated rats. A, Schematic diagram for pharmacological inhibition of GSK‐3β. Rats were pre‐treated with LiCl (0‐200 mg/kg) for 3 d and then stimulated with LPS (4 mg/kg) for 6 h prior to analyse cardiac function and harvest heart tissue. B, The down‐regulation of GSK‐3β was measured by detecting the level of β‐catenin in CMs with Western blot. C, Echocardiography assays for left ventricular fractional shortening (%) and ejection fraction (%). D, Haematoxylin‐eosin (HE) staining for myocardial tissue (Scale bar: 50 μm). E, Wheat germ agglutinin staining (WGA) for myocardium (Scale bar: 25 μm; n = 5). F, TUNEL staining assay for apoptosis (n = 5; Scale bar: 75 μm). G, H, Western blot analysis for GSK‐3β, β‐catenin, FOXO3A, Bim and Bcl‐2. I, qRT‐PCR analysis for the cytokines TNF‐α, IL‐1β, IL‐6 and iNOs (n = 4). *P < .05; **P < .01; ***P < .001 and ****P < .0001 when compared with controls

To evaluate the in vivo role of GSK‐3β, LPS (4 mg/kg) was intraperitoneally administrated into rats to induce septic myocardial dysfunction as previously described,^12^ and LiCl was also applied to inhibit the activity of GSK‐3β for three consecutive days at different concentrations (50, 100 and 200 mg/kg) before or after LPS treatment (Figure 3A). The up‐regulation of β‐catenin in heart suggested an inhibition role on GSK‐3β (Figure 3B). Lipopolysaccharide significantly impaired heart function, including decrement ejection and shortening fraction, while GSK‐3β inhibition could improve the EF by 13.85% and FS by 12.22% in the rats (Figure 3C; Figure S1). Myocardium damage assay demonstrated that GSK‐3β inhibition not only attenuated the LPS‐induced injury in hearts (Figure 3D) but, meanwhile, could partially alleviate the change in the cell size in LPS‐treated CMs (Figure 3E). However, no obvious effect on cardiac dimensions was observed in the condition (Figure S2).

TUNEL staining confirmed that GSK‐3β inhibition also reduced apoptotic cells in heart challenged with LPS (Figure 3F). Consistent with experiments in vitro, LPS could increase the expression level of GSK‐3β, FOXO3A and Bim, which could be reversed when exposed to LiCl (Figure 3G,H). In addition, the GSK‐3β inhibition suppressed the LPS‐induced activation for pro‐inflammatory cytokines IL‐6, IL‐1β, TNF‐α and iNOs in vivo (Figure 3I). Although left ventricular function was reduced, no obvious cardiac fibrosis was detected in LPS‐treated rats by Masson's trichrome staining assay even the corresponding genes expression signatures revealed a decrease in fibrosis markers (Col1a, Col3a, Fibronectin and α‐SMA), which could be partly reversed by GSK‐3β inhibition (Figure S3). These results suggest that the block of GSK‐3β could significantly improve cardiac function and attenuate apoptosis in LPS‐treated rats.

### 3.4 Inhibition of β‐catenin aggravates the LPS‐induced cell apoptosis

**Figure 4:**
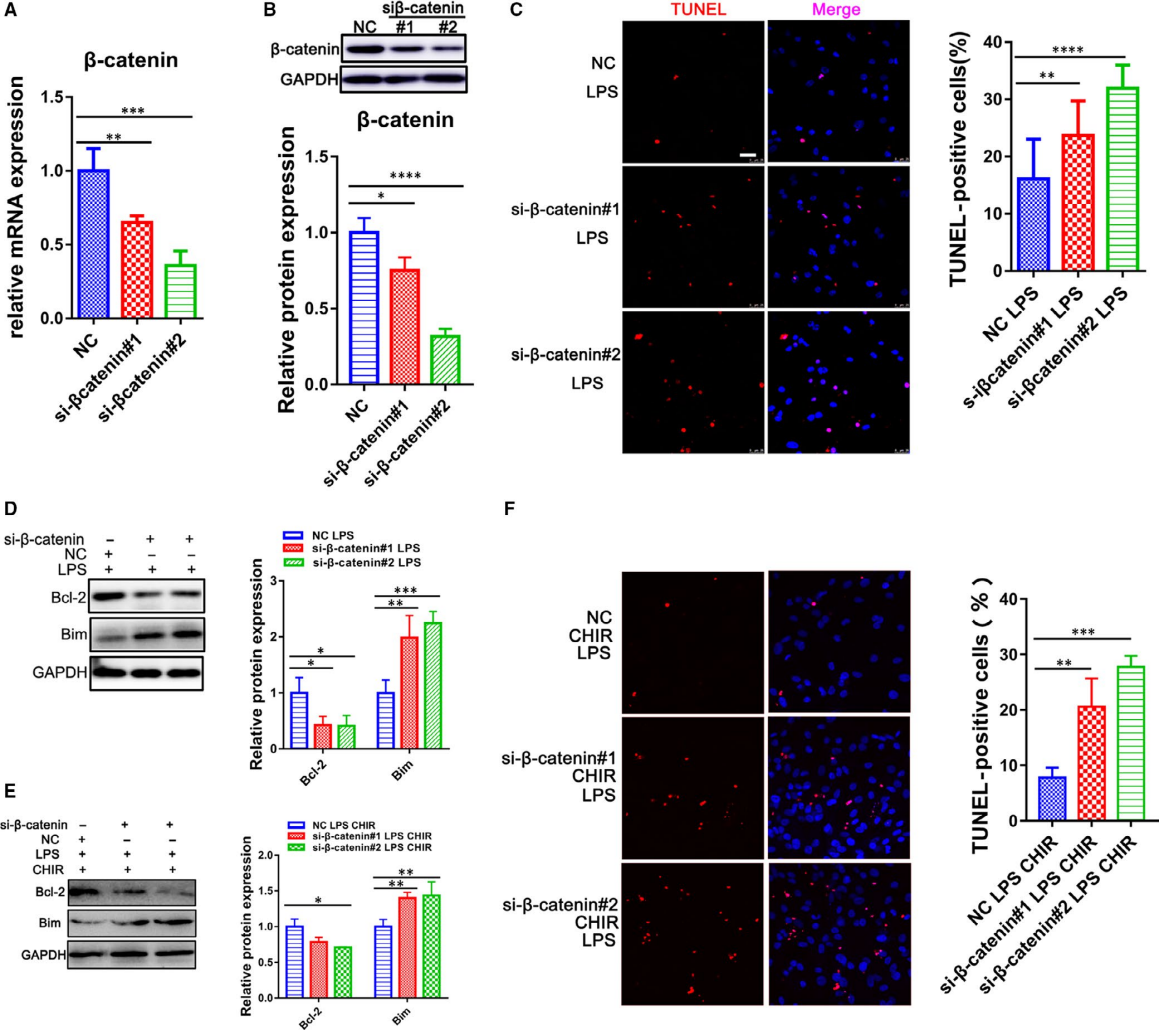
β‐catenin mediates the effects of GSK‐3β on apoptosis of cardiomyocytes. A, B, qRT‐PCR and Western blot for expression level of β‐catenin (n = 3). C, Cell death in CMs was assessed by TUNEL staining (Scale bar: 25 μm). The right panel is the percentage of TUNEL‐positive cells (n = 3). D, E, Western blot analysis for Bcl‐2 and Bim under different conditions (n = 3). F, Cell death in CMs was assessed by TUNEL staining. (Scale bar: 25 μm). The right panel is the percentage of TUNEL‐positive cells (n = 3). *P < .05; **P < .01; ***P < .001 and ****P < .0001 when compared with controls

β‐catenin is the major downstream transcription factors and proteolyzed by the destruction complex containing GSK‐3β.^10^ To determine the role of β‐catenin on the GSK‐3β regulation in LPS‐induced apoptosis of CMs, the specific siRNAs were transfected into CMs to inhibit β‐catenin expression (Figure 4A,B). We treated CMs with LPS, two siRNAs that targeted β‐catenin, and Wnt/β‐catenin inhibitors, XAV939 and ICG‐001. The knock‐down of β‐catenin really attenuated the Bcl‐2 and increased Bim expression after LPS exposure (Figure 4D; Figure S4). Meanwhile, TUNEL‐positive cells were also significantly increased under the condition (Figure 4C; Figure S4). Then, we exposed CMs to β‐catenin siRNAs as treated with GSK‐3β inhibitor, CHIR‐99021. The results shown that the effect of GSK‐3β inhibition on sepsis was prevented by the knock‐down of β‐catenin (Figure 4E,F), indicating that β‐catenin exerts an important role in GSK‐3β related sepsis.

### 3.5 GSK‐3β inhibition decreases FOXO3A expression mediated by β‐catenin

**Figure 5:**
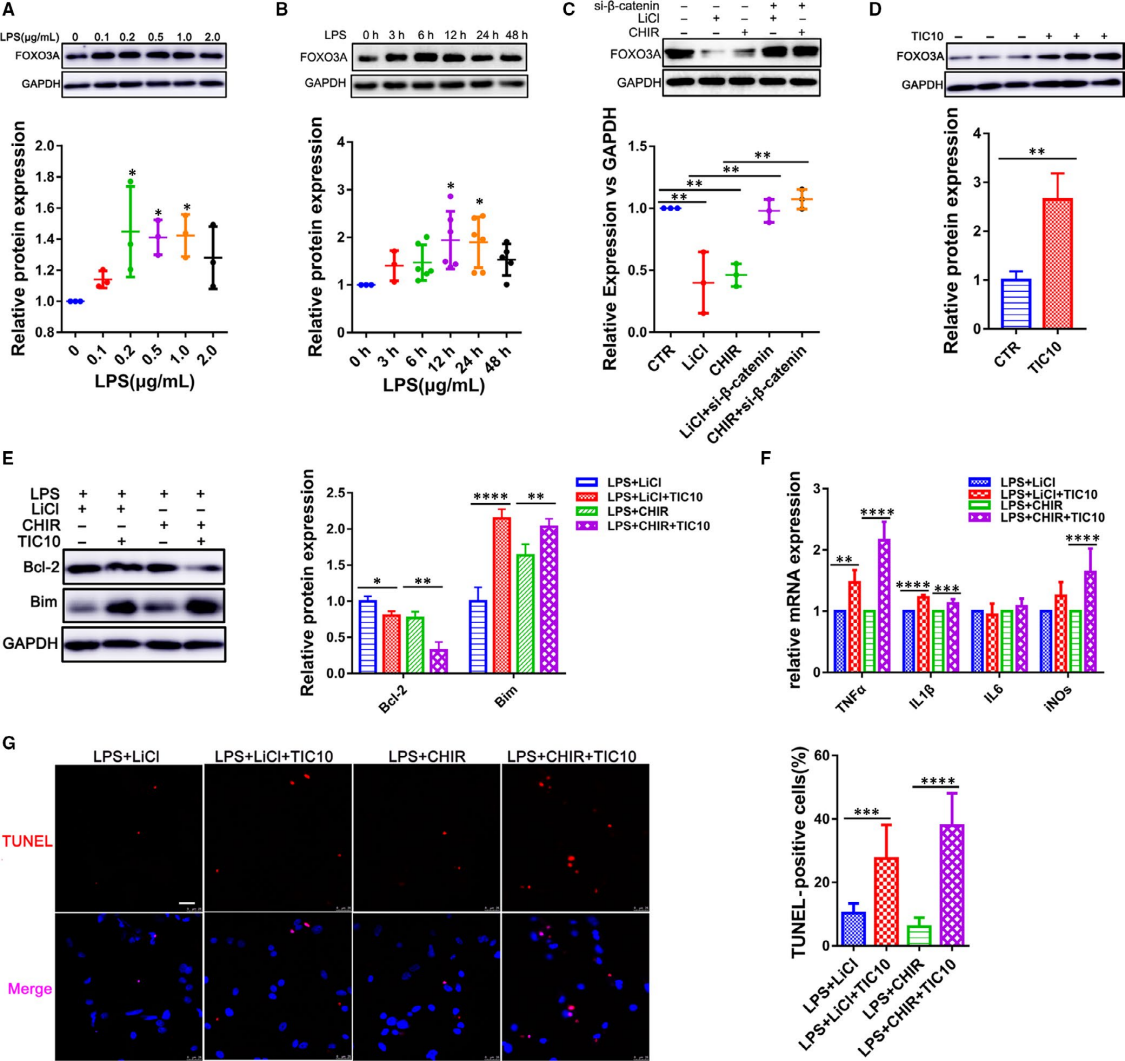
FOXO3A mediates the effects of GSK‐3β on the inflammation injury of cardiomyocytes. A, B, Immunoblots analysis for FOXO3A in CMs treated with LPS at different concentrations and time (n = 3). C, Western blot assay for FOXO3A in CMs transfected with β‐catenin for 24 h, followed by the treatment with LiCl (10 mM) or CHIR‐99021 (10 μM) for another 24 h (n = 3). D, Western blot for FOXO3A in CMs treated with TIC10 (10 μM) for 24 h (n = 3). E, Western blot for detecting the expression of Bcl‐2 and Bim. F, The mRNA expression level of pro‐inflammatory cytokines TNF‐α, IL‐1β, IL‐6 and iNOs in CMs treated with LiCl (10 mM), CHIR‐99021 (10 μM) and TIC10 (10 μM) in the presence of LPS (500 ng/mL) for 24 h (n = 3), and apoptosis of the CMs was detected by (G) TUNEL staining. The right panel is the percentage of TUNEL‐positive cells (n = 3). (Scale bar: 25 μm). *P < .05; **P < .01; ***P < .001 and ****P < .0001 when compared with controls

We next examined whether the activation of LPS‐induced inflammation injury by GSK‐3β was involved in the regulation of FOXO3A signalling. Lipopolysaccharide treatment with different concentrations (0.1, 0.2, 0.5, 1.0 and 2.0 μg/mL) and time‐points (0, 3, 6, 12, 24 and 48 hours) could significantly increase the FOXO3A expression (Figure 5A,B; Figure S5), in which FOXO3A expression level reached a peak after 12‐hour exposure with 500 ng/mL in CMs. To prove our hypothesis that FOXO3A might be one of the targets of GSK‐3β in sepsis‐induced cardiac dysfunction, we used CHIR‐99021 and LiCl to inhibit GSK‐3β. As indicated in Figure 5C; Figure S3, expression of both FOXO3A and its target gene LC3 were decreased along with inhibition of GSK‐3β (Figure S5). Moreover, the inhibition of FOXO3A could be rescued by knock‐down of β‐catenin through transfecting siRNA (Figure 5C). Then, we further examined whether FOXO3A was responsible for the protection of inflammation injury in the CMs induced by the GSK‐3β inhibition. We treated CMs with the GSK‐3β inhibitors (CHIR‐99021 and LiCl) in the presence and absence of the FOXO3A activator TIC10 and exposed them to LPS. We found that TIC10 really increased the FOXO3A level (Figure 5D), with an up‐regulation of inflammatory cytokines IL‐1β, TNF‐α and iNOs as well (Figure 5F). In addition, TUNEL staining and Western blot analysis for Bcl‐2/Bim showed that TIC10 significantly enhanced the apoptosis of the CMs (Figure 5E,G), indicating that the FOXO3A as one of the target of the GSK‐3β could regulate endotoxemia cardiac dysfunction which might attribute to its pro‐apoptotic effect on the CMs.

### 3.6 FOXO3A knock‐down attenuates LPS‐induced cardiac inflammation injury

**Figure 6:**
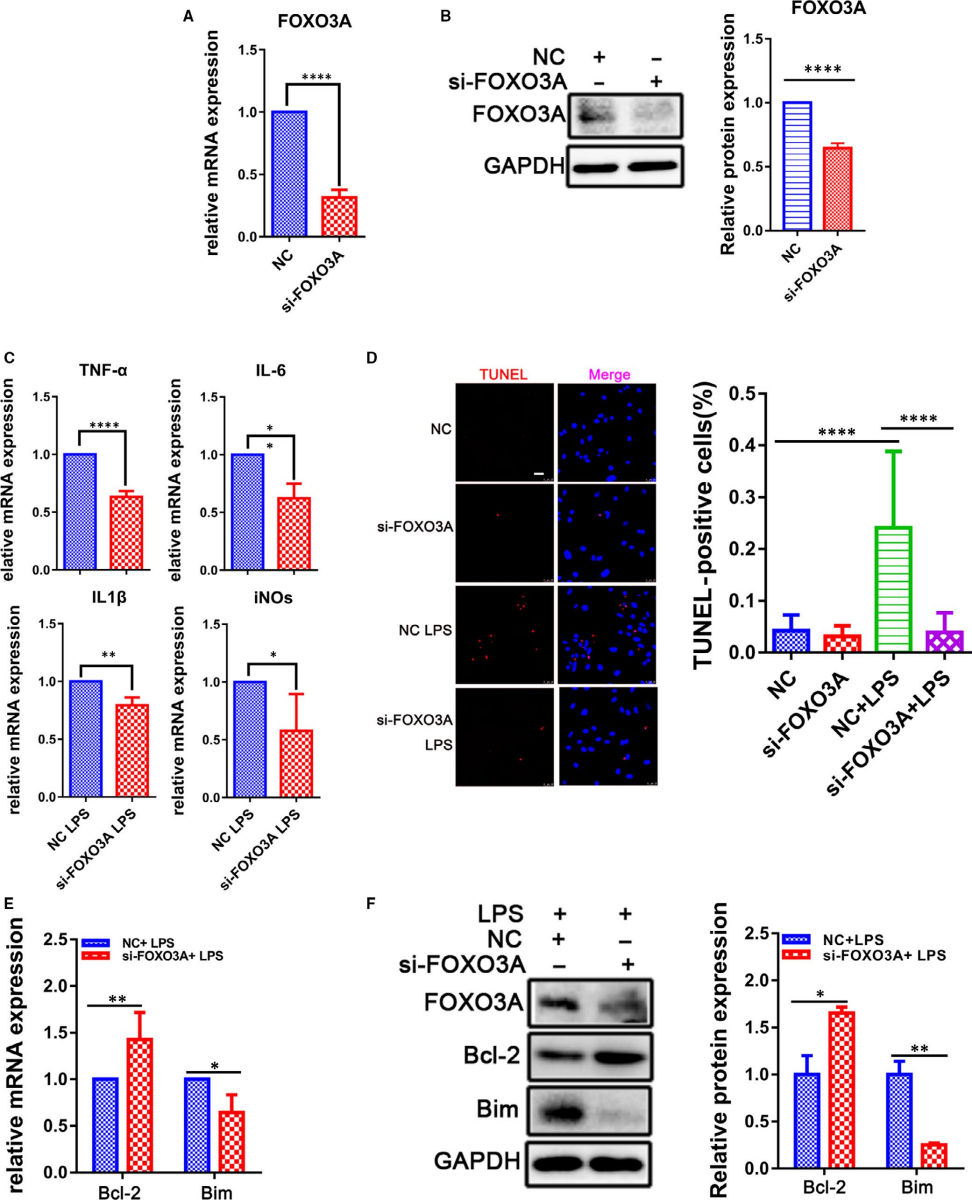
FOXO3A knock‐down attenuates LPS‐induced myocardial inflammatory injury. A, B, qRT‐PCR and Western blot for FOXO3A in CMs transfected with siRNA for 48 h (n = 3). C, The mRNA levels of pro‐inflammatory cytokines TNF‐α, IL‐1β, IL‐6 and iNOs were measured by qRT‐PCR in CMs transfected with FOXO3A siRNA and stimulated with LPS (500 ng/mL) for another 12 h (n = 3). D, TUNEL staining and E, F, qRT‐PCR and Western blot for Bcl‐2 and Bim of CMs pre‐treated with si‐FOXO3A for 24 h and challenged with or without LPS (500 ng/mL) for another 12 h (n = 3). (Scale bar: 25 μm). *P < .05; **P < .01; and ****P < .0001 when compared with controls

To confirm the FOXO3A function in the LPS‐induced sepsis in CMs, knock‐down assay showed the expression level of FOXO3A was significantly decreased in the CMs transfected with the siRNA targeting FOXO3A for 48 hours (Figure 6A,B), in which the LPS‐induced up‐regulation of IL‐6, IL‐1β, TNF‐α and iNOs was suppressed (Figure 6C). Meanwhile, FOXO3A knock‐down also attenuated the activation of NF‐κB (Figure S6). These results suggest that the LPS‐induced FOXO3A could regulate inflammation process in CMs. Moreover, the down‐regulation of FOXO3A could decrease the LPS‐induced apoptotic index (Figure 6D), accompanying with an increase in Bcl‐2 and a decrease in Bim. All of the data indicated that FOXO3A was associated with the endotoxin‐induced myocardial apoptosis (Figure 6E,F) and played a crucial role in the inflammation injury of the heart.

### 3.7 GSK‐3β inhibition negatively involved in the activations of ERK/NF‐κB signalling and positively regulated AMPK pathway

**Figure 7:**
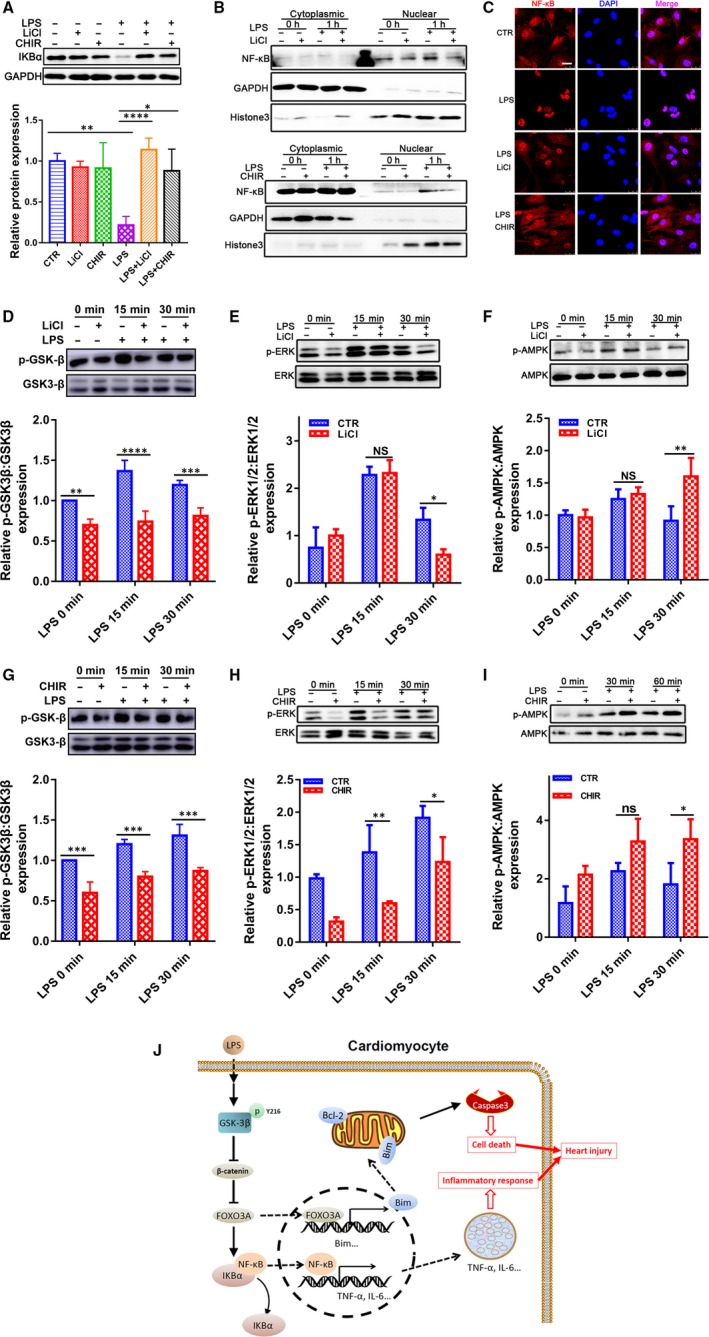
GSK‐3β inhibition inversely correlates with activation of ERK and NF‐κB pathways and positively related with activations of AMPK signalling. A, Western blot analysis for IKBα (n = 3). B, C, Western blot for nuclear proteins and immunofluorescence staining for CMs treated with GSK‐3β inhibitors with or without LPS (n = 3). (Scale bar: 25 μm). Phosphorylation of GSK‐3β, ERK1/2 and AMPK was measured by Western blot in CMs treated with GSK‐3β inhibitors, D‐F, LiCl (10 mM) or G‐I, CHIR‐99021 with or without LPS (n = 3). *P < .05; **P < .01; ***P < .001 and ****P < .0001 when compared with controls. J, Graphic summary. FOXO3A is transcriptionally activated by LPS in CMs through the activation of GSK‐3β/β‐catenin pathway and regulates LPS‐induced heart damage by targeting Bim and NF‐κB

IκBα is degraded upon phosphorylation which results in the consequent release and nuclear translocation of NF‐κB.^27^ When cells were stimulated with LPS, the degradation of IκBα induced an increase in NF‐κB level in the nuclear fraction (Figure 7A‐C). However, GSK‐3β inhibitors LiCl or CHIR‐99021 increased the IκBα level and reduced nuclear NF‐κB only under LPS condition (Figure 7A‐C). The results suggest that NF‐κB is involved in the effect of GSK‐3β on regulating inflammation injury in heart.

In addition, ERK and AMPK signalling are two important pathways activated by LPS in many cell types. ERK contributes to the induction of inflammation and apoptosis, whereas AMPK exerts reverse effects in the processes through cross‐talking with NF‐κB.^28^ As shown in Figure 7D‐I, ERK and AMPK signalling were activated by the exposure of CMs to LPS for 15 or 30 minutes. GSK‐3β inhibition suppressed the LPS‐induced ERK1/2 phosphorylation and enhanced the LPS‐induced AMPK phosphorylation with different extent, suggesting a crucial role of GSK‐3β in inflammation injury.

## 4 DISCUSSION

In the current study, we showed that GSK‐3β was associated with cardiac dysfunction during sepsis, and its inhibition attenuated the LPS‐induced inflammation injury mediated by negatively regulating ERK/NF‐κB pathway and promoted AMPK signalling in LPS‐induced sepsis. GSK‐3β positively regulated the expression of FOXO3A via degrading β‐catenin in endotoxemia cardiac dysfunction. In addition, we showed that FOXO3A was also involved in the regulation of the inflammation injury challenged by LPS. Thus, GSK‐3β plays an important role in advancing endotoxin‐induced cardiac dysfunction, possibly via regulating FOXO3A signalling through degrading β‐catenin.

The sepsis‐induced release of enormous amounts of endotoxins such as LPS trigger a cascade of pro‐inflammatory cytokines, including IL‐1β, IL‐6, IL8 and TNF‐α, which are widely used as apoptosis‐inducing factors for in CMs.^10^, ^29^ Among the cytokines, TNF‐α can induce apoptosis in rat heart, and TNF‐α, secreted by both macrophages and CMs in sepsis, elevates the expression of various complement factors, nitric oxide (NO) synthase, cellular adhesion molecules, platelet‐activating factor and a number of interleukins, thereby playing a key role in the early pathogenesis of sepsis.^11^ IL‐1β and IL‐6 are considered as predictive markers of the development and severity for sepsis and septic shock. Moreover, mitochondrial NO has been reported to be involved in myocardial depression.^30^ In addition, GSK‐3β regulates TLR‐mediated promotion of pro‐inflammatory cytokines production and reduces anti‐inflammatory cytokines.^26^ GSK‐3β inhibitor SB216763 protects mice against endotoxin lethality induced by LPS, and Wnt inhibitor XAV 939, which usually promotes GSK‐3β activation and β‐catenin reduction, partly neutralized ADMSC‐ex–induced anti‐apoptotic and pro‐survival effects in ischaemia/reperfusion (I/R).^10^, ^31^ However, other studies show Wnt inhibitors Dickkopf‐1, LGK974 and iCRT3 attenuate LPS‐induced inflammatory response in different cells and lung injury.^32^, ^33^ These findings suggest that GSK‐3β is an important regulator for the balance between pro‐ and anti‐inflammatory cytokines.^26^, ^34^ In the work, inhibition of GSK‐3β really reduced inflammatory mediators such as TNF‐α, IL‐1β, IL‐6 and iNOs in CMs.

Apoptosis of CMs has been showed to contribute to inflammation injury in heart.^25^ Using TUNEL assay, we demonstrated that LPS could induce apoptosis in the CMs, and GSK‐3β inhibition effectively decreased the number of apoptotic cells. In addition, knock‐down of β‐catenin could aggravate LPS‐induced damage of CMs. GSK‐3β and β‐catenin are important moderators in the Wnt signalling pathway, which can both positively and negatively affect cell fate under a variety of challenge.^35^ The reduction in β‐catenin via Wnt inhibition prevents cardiomyocyte apoptosis and contractile dysfunction in acute myocardial infarction.^36^ However, activation of Wnt/β‐catenin signalling prevents ischaemia/reperfusion‐induced cardiomyocyte apoptosis by exosomes from adipose‐derived mesenchymal stem cells and protects hippocampal neurons against the apoptosis caused by oxygen‐glucose deprivation/reoxygenation.^31^, ^37^ These results suggest that Wnt/β‐catenin exert their functions as a context‐dependent manner.

FOXOs mediates the process of cell fate through transcriptionally activating or inhibiting several target genes or by cross‐talking with other factors,^38^, ^39^, ^40^ and GSK‐3β could, on other hand, transcriptionally promote the expression of FOXO3A.^18^ Here, we showed that GSK‐3β inhibition decreased FOXO3A level, which was reversed by β‐catenin knock‐down, proving that GSK‐3β could modulate FOXO3A through degrading β‐catenin. This finding is consistent with previous report that β‐catenin interacts with FOXO and enhances FOXO transcriptional activity in mammalian cells.^41^ However, activation of Wnt/β‐catenin represses murine liver oxidative stress‐induced apoptosis by inhibiting FOXO3A through targeting SGK1.^42^ In addition, FOXO3A could also regulate β‐catenin transcriptional activity through competing with TCF.^43^ These results mean that β‐catenin modulation on FOXO3A shows cell‐type specific with an animal model‐dependent pattern. Furthermore, β‐catenin has been showed to be also involved in down‐regulating FOXO3A through promoting Akt activity,^44^ which is consistent with our results. Actually, the roles of FOXO3A in heart injury so far still remain controversial. FOXO3A protects the heart from pathological hypertrophy and improves cardiac function in ischaemia‐reperfusion and hypertensive cardiac injury models.^45^ On the other hand, FOXO3A leads to organ injury via promoting cell apoptosis through transcriptional activation of pro‐apoptosis genes, such as Bim, PUMA and Mxi1‐0.^19^, ^21^ To understand the involvement of FOXO3A in the LPS‐induced cardiac dysfunction, we performed knock‐down experiments using siRNAs and observed that the down‐regulation of FOXO3A could significantly suppress the expression of pro‐inflammatory cytokines and the apoptosis. FOXO3A activator TIC10 could rescue the suppressive effects of GSK‐3β inhibitors on LPS‐induced pro‐inflammatory cytokine expression and apoptosis. Overall, these data suggest that GSK‐3β‐mediated myocardial dysfunction by LPS is controlled by the expression level of FOXO3A.

Lipopolysaccharide could trigger a series of cell signalling cascade including the NF‐κB pathway, which is crucial in the inflammatory response. NF‐κB has been demonstrated to be involved the increase in morbidity and mortality in sepsis, and its activation occurs upon the degradation of IκBα and phosphorylation of NF‐κB p65 subunit.^27^ Alike in the present study, NF‐κB p65 subsequently translocates from the cytoplasm into the nucleus and induces the expression of inflammatory mediators. Therefore, it is possible that blockade of NF‐κB activation may attenuate the sepsis‐induced myocardial dysfunction. Extracellular signal‐regulated kinases (ERKs) pathway represents one subgroup of the mitogen‐activated protein (MAP) kinases. Several studies have reported that MAP kinases are involved in the production of LPS‐induced inflammatory mediators, including NO, COX and cytokines.^46^ AMPK activation generally elaborates a potential anti‐inflammatory effect in vitro and in vivo.^8^ Like previous findings,^11^ our study demonstrates that inhibition of GSK‐3β activity via pharmacological inhibitors could reverse pro‐inflammatory signalling through blocking NF‐κB and ERK 1/2 pathways. In addition, GSK‐3β inhibition could enhance AMPK signalling activity, resulting in a suppression on the LPS‐induced heart injury.

In summary, our research shows that GSK‐3β is excessively activated in endotoxin‐induced cardiomyopathy. Pharmacological inhibition of GSK‐3β improves cardiac function and reduces apoptosis in septic hearts. These results provide new insights into the cross‐talking among GSK‐3β/β‐catenin/FOXO3A pathways to lead to inflammatory gene expression in CMs and especially under in vivo conditions, suggesting a potent therapeutic target for anti‐septic myocardial dysfunction.

## CONFLICT OF INTEREST

The authors confirm that there is no conflict of interest.

## AUTHORS' CONTRIBUTIONS

L Peng and L Li conceived and supervised the project; Z Li designed and performed the experiments. Z Li and C Liu analysed the data; H Zhu, D Wang, H Liu and W Cao carried out part of experiments; Y Wang, Y Hu, Q Lin, C Tong and M Lu were involved in the discussion of results. Z Li wrote the manuscript. L. Peng, A. Sachinidis and C. Liu revised the manuscript. All authors read and approved the manuscript.

## Data Availability

The data that support the findings of this study are available from the corresponding author upon reasonable request.
