# Supplementary figures and images for: GSK‐3β inhibition protects the rat heart from the lipopolysaccharide‐induced inflammation injury via suppressing FOXO3A activity
Source: J Cell Mol Med. 2019 Sep 10;23(11):7796–809. doi: 10.1111/jcmm.14656 (PMC6815822; doi:10.1111/jcmm.14656)

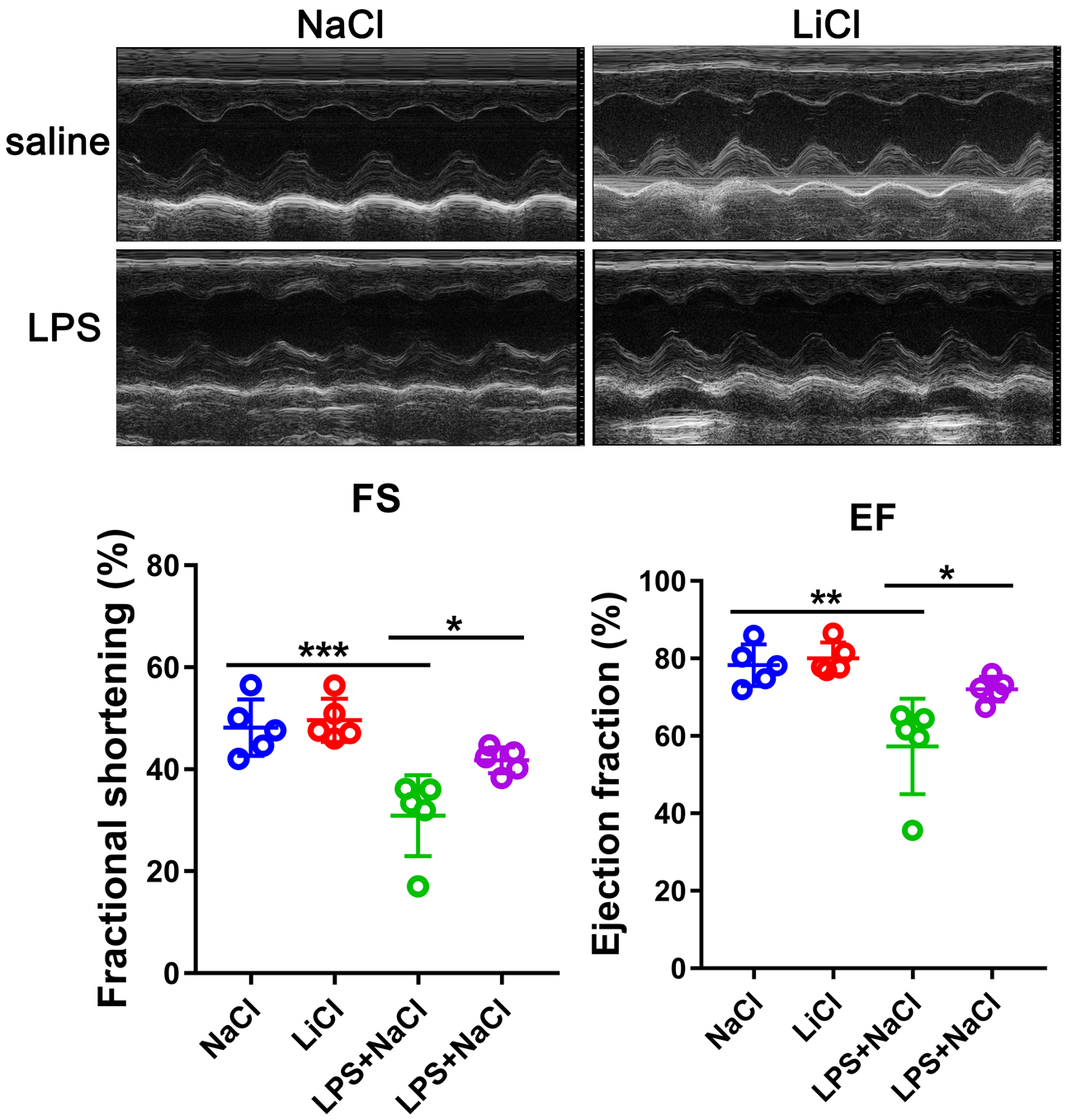

Supplement: Supplementary file 1 [file JCMM-23-7796-s001.tif]

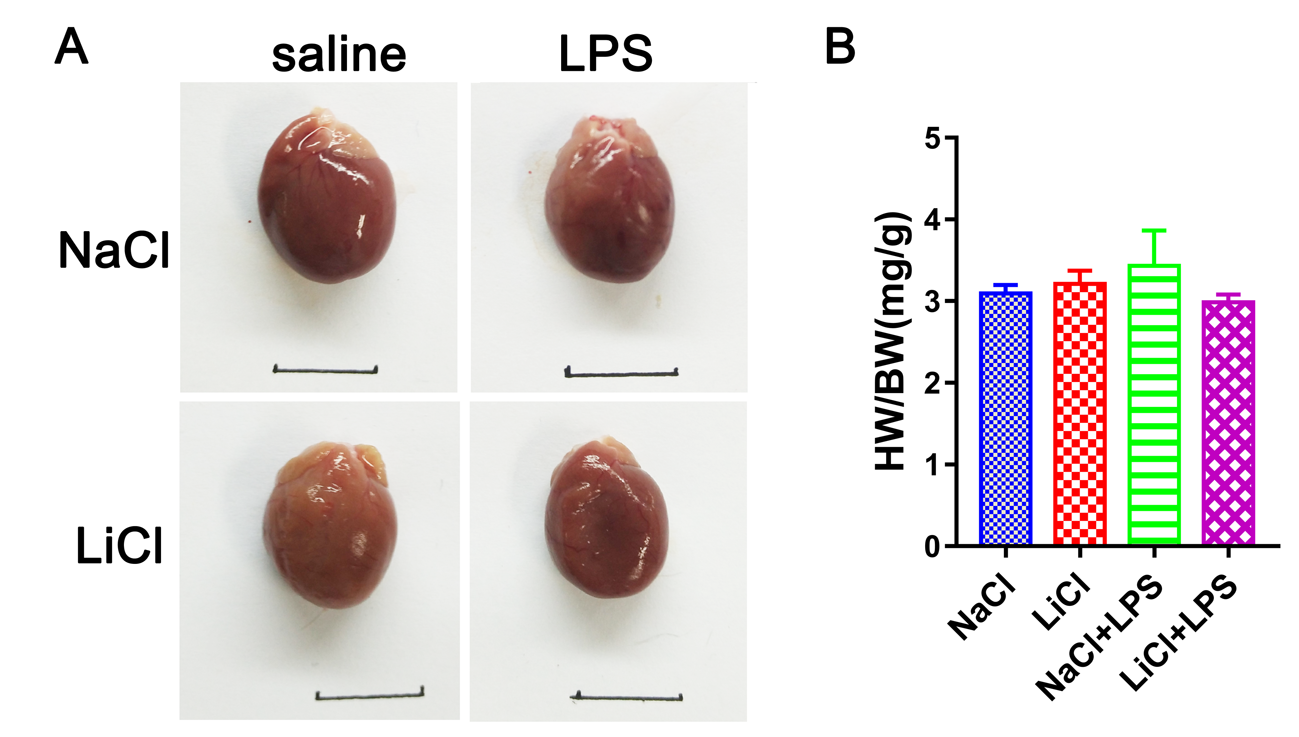

Supplement: Supplementary file 2 [file JCMM-23-7796-s002.tif]

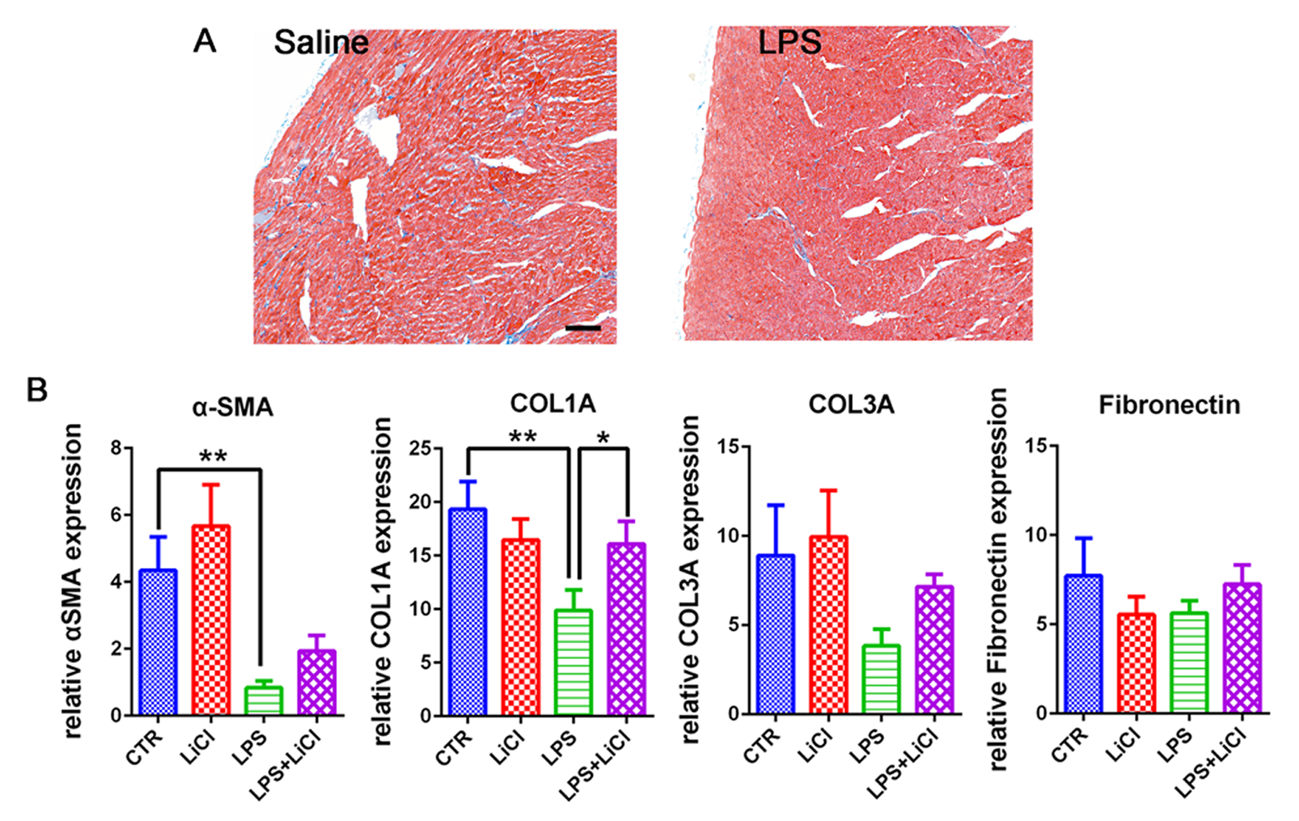

Supplement: Supplementary file 3 [file JCMM-23-7796-s003.tif]

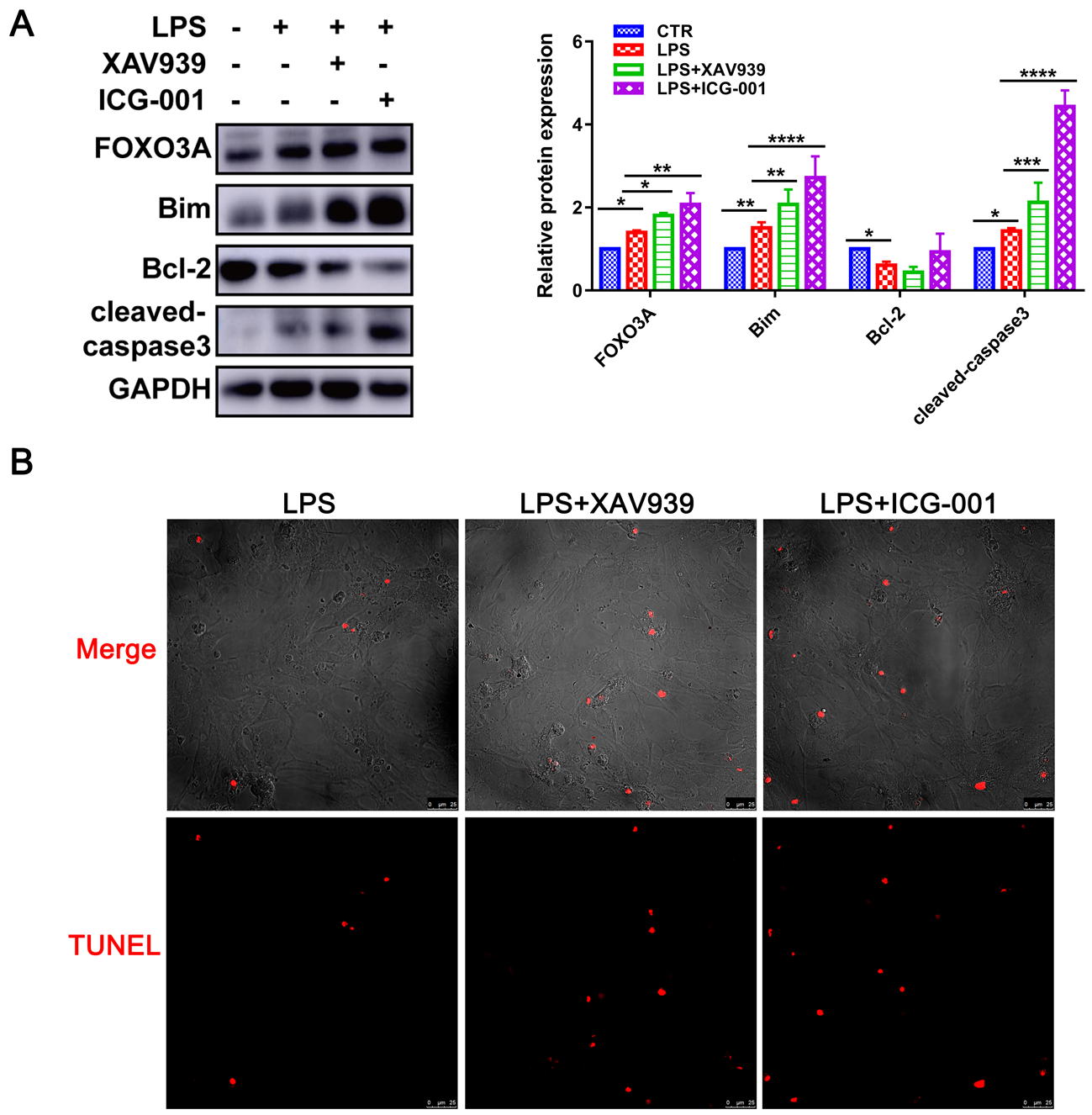

Supplement: Supplementary file 4 [file JCMM-23-7796-s004.tif]

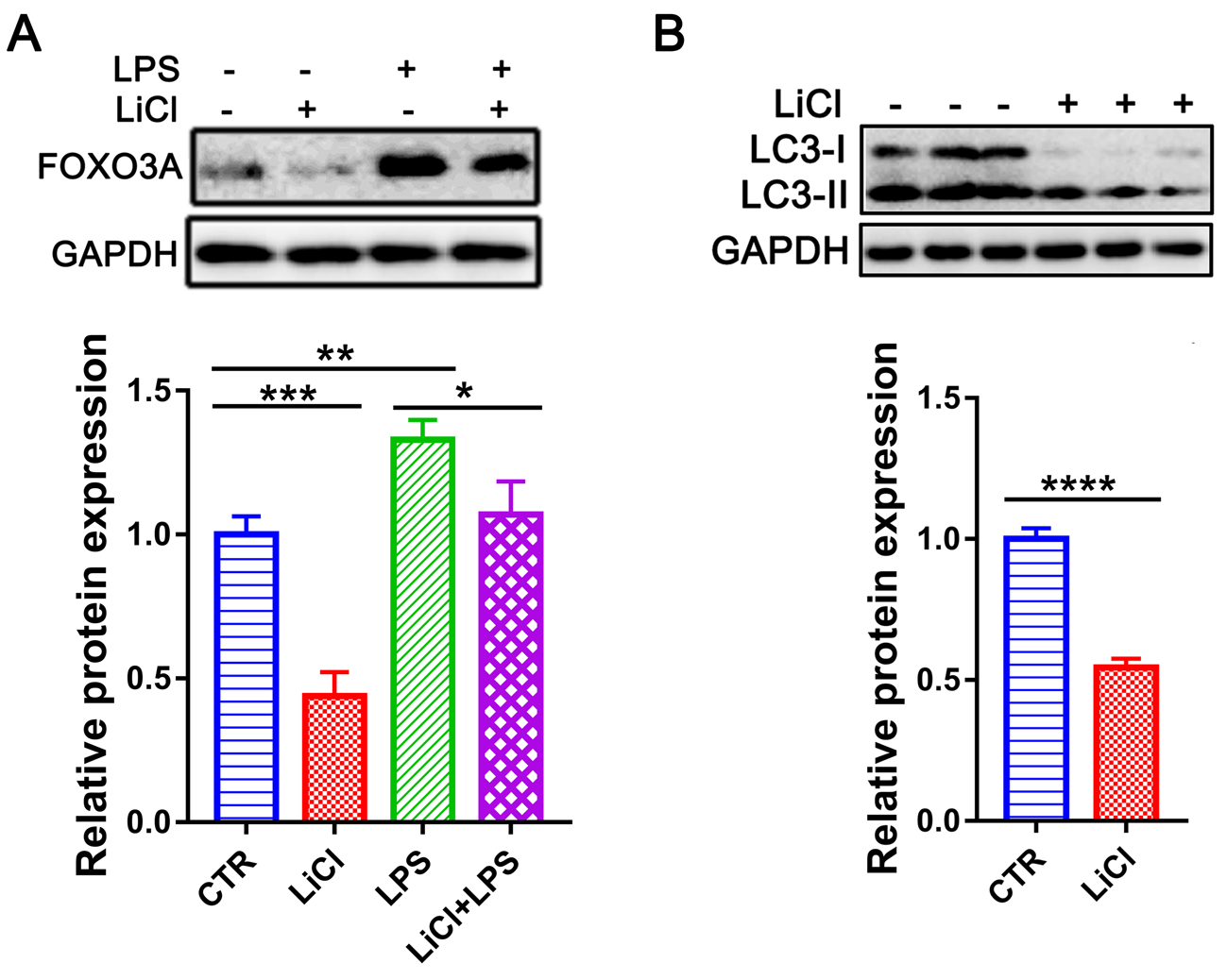

Supplement: Supplementary file 5 [file JCMM-23-7796-s005.tif]

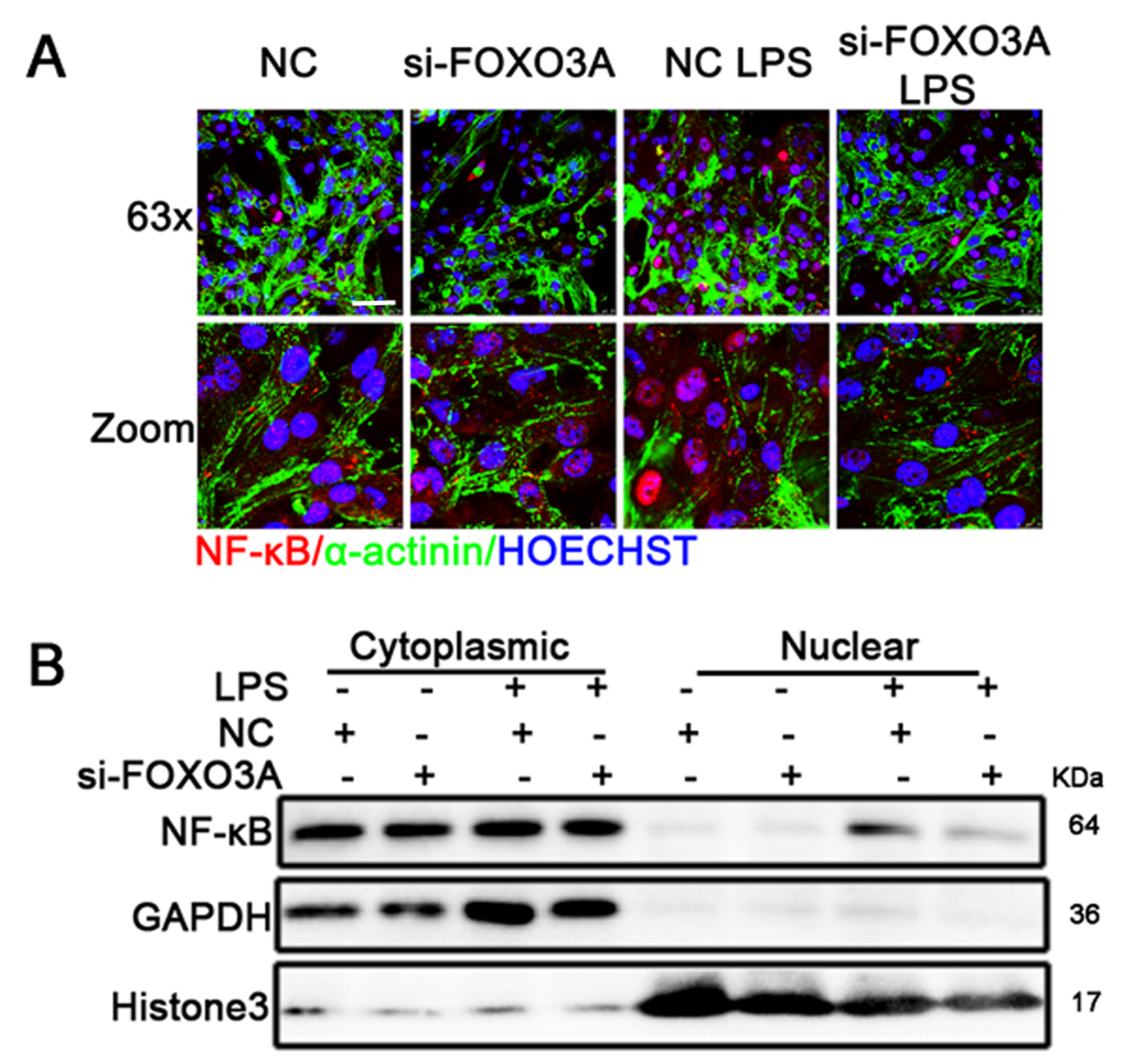

Supplement: Supplementary file 6 [file JCMM-23-7796-s006.tif]
