# Supplementary material for: GSK‐3β inhibition protects the rat heart from the lipopolysaccharide‐induced inflammation injury via suppressing FOXO3A activity
Source: J Cell Mol Med. 2019 Sep 10;23(11):7796–809. doi: 10.1111/jcmm.14656 (PMC6815822; doi:10.1111/jcmm.14656)
Supplement: Supplementary file 7 [file JCMM-23-7796-s007.docx]

**Supplemental figure legends**

**Figure_S1 GSK-3β inhibitor LiCl improves cardiac function after LPS challenge**

(A) Echocardiography assays of Left ventricular fractional shortening (%) and ejection fraction (%) after being treated with LPS(4mg/kg) and followed with LiCl (100mg/kg) through Intraperitoneal injection (IP) for 6h in rats.

**Figure_S2 The effects of GSK-3β inhibitors on cardiac dimensions**

(A-B) Heart weight corrected for body weight, (Scale bar: 1.0 cm).

**Figure_S3 Down-regulation of GSK-3β affected cardiac fibrosis in LPS-treated rats**

(A) Representative myocardial sections stained for fibrosis with Masson’s Trichrome staining induced by LPS(500 ng/mL). (Scale bar: 100 μm). (B) The mRNA expression levels of fibrosis markers genes Col1a, Col3a, Fibronectin and α-SMA were measured by qRT-PCRs in Cardiac fibroblasts(CFs) treated with LiCl (10mM) under challenging of LPS (500ng/ml) for 12h(n=3). *, P<0.05; **, P<0.01 compared with control; Unpaired Student’s t-test was used to evaluate the statistical significance.

**Figure_S4 Wnt/β-catenin inhibitors aggravated apoptosis induced by LPS**

(A) Western blotting analysis of FOXO3A, Bcl-2, Bim and cleaved-caspase3 treated with XAV-939 or ICG-001, and stimulated with LPS (500ng/ml) for another 12h. (B) Cell death in CMs was assessed by TUNEL staining (Scale bar: 25 μm). after β-catenin. *P < 0.05; **P < 0.01, ***P < 0.001 and ****P<0.0001 compared with control

**Figure_S5 Inhibition of GSK-3β reduces FOXO3A activity**

(A-B)Immunoblots analysis of FOXO3A and LC3 (c) in CMs treated with LiCl (10mM) under challenging of LPS (500ng/ml) for 24h. (n=3). Unpaired Student’s t-test was used to evaluate the statistical significance.

**Figure_S4 FOXO3A knockdown decreased nuclear amount of NF-κB induced by LPS**

(A)Immunofluorescence staining and (b) Nuclear western blot from CMs transfected with FOXO3A for 48h and then stimulated with or without LPS (500 ng/mL) for another hour noted that NF-κB expression was retained in the cytoplasm in GSK-3β inhibition CMs despite LPS treatment(n=3). (Scale bar: 25 μm). Unpaired Student’s t-test was used to evaluate the statistical significance.
