# Supplementary material for: GSK‐3β inhibition protects the rat heart from the lipopolysaccharide‐induced inflammation injury via suppressing FOXO3A activity
Source: J Cell Mol Med. 2019 Sep 10;23(11):7796–809. doi: 10.1111/jcmm.14656 (PMC6815822; doi:10.1111/jcmm.14656)
Supplement: Supplementary file 8 [file JCMM-23-7796-s008.docx]

| **Gene** | **Species** | **Forward primer** | **Reverse primer** |
| --- | --- | --- | --- |
| TNF-α | rat | ATCCGAGATGTGGAACTGGC | CGATCACCCCGAAGTTCAGT |
| iNOs | rat | ACGCTACACTTCCAACGCA | ACACGTTCTTGGCGTGGAT |
| IL-1β | rat | CTTGACTTGGGCTGTCCAGA | ACGGGCAAGACATAGGTAGC |
| IL-6 | rat | AGAGACTTCCAGCCAGTTGC | AGTCTCCTCTCCGGACTTGT |
| Bim | rat | TCCGATCGGCACAACACG | GTCGGGATTACCTTGCGATTC |
| Bcl-2 | rat | AGCATGCGACCTCTGTTTGA | TCACTTGTGGCCCAGGTATG |
| β-catenin | rat | ACTCCAGGAATGAAGGCGTG | GAACTGGTCAGCTCAACCGA |
| FOXO3A | rat | CGGCTCACTTTGTCCCAGAT | TTCGTTCTGAACCCGCATGA |
| GAPDH | rat | AAGGTCGGTGTGAACGGATT | CTTTGTCACAAGAGAAGGCAGC |

**Table S1** Primer sequences used in this study
